# Characterization of occult hepatitis B infection among Iranian liver transplant recipients

**DOI:** 10.1002/jcla.24614

**Published:** 2022-09-09

**Authors:** Azam Khamseh, Vahdat Poortahmasebi, Saber Soltani, Mohsen Nasiritoosi, Ali Jafarian, Azam Ghaziasadi, Mehdi Norouzi, Saied Ghorbani, Narges Eslami, Seyed Mohammad Jazayeri

**Affiliations:** ^1^ Research Center for Clinical Virology Tehran University of Medical Sciences Tehran Iran; ^2^ Department of Virology School of Public Health Tehran University of Medical Sciences Tehran Iran; ^3^ Infectious and Tropical Disease Research Center Tabriz University of Medical Sciences Tabriz Iran; ^4^ Department of Bacteriology and Virology, School of Medicine Tabriz University of Medical Sciences Tabriz Iran; ^5^ Liver Transplantation Research Center Tehran University of Medical Sciences Tehran Iran; ^6^ Department of Virology Iran University of Medical Sciences Tehran Iran

**Keywords:** HBV reactivation, liver transplantation, occult HBV infection

## Abstract

**Background:**

The prevalence of occult hepatitis B infection (OBI) among Iranian liver transplant recipient patients has not been explored yet. The present study aimed to determine the OBI prevalence among Iranian liver transplant recipients.

**Methods:**

This study encompassed 97 patients having undergone liver transplantation due to several clinical backgrounds in the Liver Transplantation Center, Tehran, Iran. After serological evaluation, two different types of PCR methods were applied for amplification of HBV DNA, followed by the direct sequencing of whole hepatitis B virus (HBV) surface genes.

**Results:**

At the time of admission, none of the patients were positive for HBsAg. However, 24 (25%), 12 (12.3%), and 5 (5.1%) cases were positive for anti‐HBc, hepatitis C virus (HCV), and hepatitis delta virus (HDV) antibodies, respectively. Moreover, two males were positive for OBI (2.1%). Both were positive for anti‐HBc and negative for anti‐HBs, anti‐HCV, and anti‐HDV. HBV‐related cirrhosis was the underlying reason for their liver transplantation. HBsAg sequences revealed no amino acid substitution.

**Conclusions:**

The prevalence of OBI in the Iranian liver transplantation patients was relatively low. Future longitudinal studies with a larger sample size are suggested to explore the significance of this clinical finding, including the reactivation of cryptic HBV DNA, in liver transplant subjects.

## Graphical abstract

The aim of this study was to characterize the prevalence of occult HBV infection in a series of liver transplant recipients. The present study comprised 97 patients who underwent liver transplantation. HBV serologic and molecular assays were performed. The hepatitis B surface antigen (HBsAg) genes from HBV‐DNA‐positive samples were amplified and directly sequenced. At the time of presentation, none were positive for HBsAg. However, 24 (25%), 12 (12.3%), and 5 (5.1%) were positive for anti‐HBc, hepatitis C virus (HCV), and hepatitis delta virus (HDV) antibodies, respectively. Two males were positive for OBI (2.1%). Both were positive for anti‐HBc and were negative for anti‐HBs, anti‐HCV, and anti‐HDV. HBV‐related cirrhosis was the underlying reason for their liver transplantation. Both OBI samples belonged to HBV genotype D and subtype awy2. HBsAg sequences did not show any amino acid substitution.

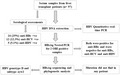

## 1 INTRODUCTION

Occult hepatitis B infection (OBI) refers to detecting HBV DNA in the absence of hepatitis B surface antigen (HbsAg). Due to its cryptic nature, OBI still retains the same pro‐oncogenic features. It may contribute to the acute exacerbation and development of hepatitis B virus (HBV) associated diseases such as liver diseases, cirrhosis, and hepatocellular carcinoma (HCC).^1^, ^2^ OBI has been described in various clinical settings,^2^ and one of the main clinical implications of OBI is usually observed in the setting of liver transplantation. In particular, livers from donors with OBI carry a risk of HBV transmission, with infection arousing in 25%‐95% of the liver grafts donated from patients being HbsAg‐negative and anti‐HBc‐positive. This infection route cannot be distinguished from the chronic HbsAg‐positive carrier state.^2^ The significance of OBI in post‐orthotopic liver transplantation settings is controversial. Upon the analysis of intrahepatic HBV DNA, some patients show high liver or serum HBV DNA levels and a high reactivation rate,^3^, ^4^ whereas some others have revealed low HBV reactivation rates.^5^, ^6^, ^7^ In this regard, the main goal is to maintain anti‐HBs above the protection level (>10 IU/mL) in vaccine recipients. In 1993, the Iranian Ministry of Health launched an HBV vaccination program in all provinces, leading to the 98% coverage of all infants across the country. It is mandatory for neonates born after this time to be vaccinated using a recombinant HBsAg with classic three consecutive doses. According to recent statistics, Iran is classified as a low HBV prevalence area (<2%), indicating the effectiveness of the preventive measures in Iran.^8^ It is postulated that OBI can be found in most recipients of livers from HBsAg‐negative and anti‐HBc‐positive donors. The likelihood of HBV reactivation would be minimized by immunization and preemptive treatment with nucleos(t)ide analog drugs as a standard strategy to prevent hepatitis B infection.^9^ Accordingly, liver transplant recipients currently receive pre‐, during, and post‐transplantation hepatitis B immune globulin (HBIG) with different options along with antivirals according to different global protocols.^10^ This study aimed to estimate the prevalence of HBsAg carriers and occult HBV infection among the Iranian liver transplant recipients.

## 2 MATERIALS AND METHODS

### 2.1 Patients

The present cross‐sectional study encompassed 97 patients undergoing liver transplantation at the Liver Transplantation Center, Imam Khomeini Hospital, Tehran, during 2005‐2018. They had the following clinical backgrounds as candidates for liver transplantation: cryptogenic cirrhosis (49, 50.6%), HBV‐related cirrhosis (13, 13.4%), HCV‐related cirrhosis (12, 12.4%), HBV/HCV‐related cirrhosis (1, 1%), cirrhosis due to autoimmune hepatitis (17, 17.6%), fulminant hepatitis (1, 1%), Budd‐Chiari (1, 1%), and primary sclerosing cholangitis (3, 3%). Most of the patients were transplanted during the last 3 years. Blood samples were collected during 2017‐2018. The study was evaluated and approved by the Ethics Committee of the Tehran University of Medical Sciences. The patients' history for the HBV vaccination before the transplantation time was unknown, and the authorities assumed that the transplant recipients had not received the HBV vaccine before transplantation. According to the routine guidelines in this center, all LT candidates received polyclonal HBIG 10000 international unit (IU) intramuscularly immediately before transplantation, followed by 5000 IU for the first seven postoperative days. Then, the patients received 1000 IU for 4 weeks and then monthly to maintain 250 IU. All HbsAg‐positive patients also received Tenofovir (300 mg daily) before transplantation and after transplantation as long as anti‐HBs achieved 300 IU. Finally, all transplant patients received daily Prograf (6 mg), Cellcept (2 mg), and Prednisolon (10 mg).

### 2.2 Serological assessments

The viral markers of HBV, including HBsAg, anti‐HBs, anti‐HBc, and anti‐hepatitis delta virus (HDV; Diaper), were measured by the enzyme‐linked immunosorbent assay according to the manufacturer's protocol.

### 2.3 DNA extraction, polymerase chain reaction, and DNA sequencing

HBV DNA was extracted from 200 μL of the aliquot of serum using the Qiagen Mini Blood Kit (Qiagen) according to the manufacturer's instruction. Regardless of the serologic results, a quantitative real‐time PCR was applied for all subjects using Fast‐track diagnostics/SIEMENS kits (Luxembourg) following the manufacturer's recommendations. The dynamic range of the assay claimed by the manufacturer was 10^2^‐10^9^ IU/mL [2‐9 log_10_] IU/mL, and the lower detection limit of the assay was 85 IU/mL. Those samples negative in primary screening by real‐time PCR were re‐checked for OBI using another HBsAg gene‐nested PCR approach, which was recommended by Taormina expert meeting on occult HBV infection.^11^ Subsequently, a nested PCR was carried out for positive cases, as described previously.^12^

The nucleotide sequences of the HBsAg encoding component were determined bilaterally by the 3130 Genetic Analyzer (Genetic Analyzer ABI‐3130 DNA Sequencer). The sequences were compared, and edited, and the extra sections were deleted with Chromas software. Finally, the sequences encompassing the 681 nucleotides of the gene encoding HBsAg were transferred to BioEdit software version 7.0.9 in FASTA format. Seven HBV genotype D‐matched sequences (namely CQ183486, AY161150, AB0335591, AF061523.1, X80925, X65259, and X65259 were obtained from the NCBI site) to study the mutations in the HBsAg region. Genotyping was performed by the phylogenetic analyses for the reference sequences of the HBV genotypes A‐H. A maximum likelihood (ML) tree was created using the alignment of the HBsAg gene of the subjects by rooting with a Woodchuck hepatitis virus. The phylogenetic tree was generated with MEGA X software, and the genetic distance was calculated using Kimura's two‐parameter matrix.^13^ Bootstrap resampling and reconstruction were carried out 1000 times to confirm the reliability of the phylogenetic tree.^14^

### 2.4 Statistical analysis

Data were analyzed with the Statistical Package for the Social Sciences (SPSS‐21, SPSS Inc.). The continuous variables were expressed as mean ± standard deviation or median, and the categorical variables were expressed as percentages.

## 3 RESULTS

**FIGURE 1:**
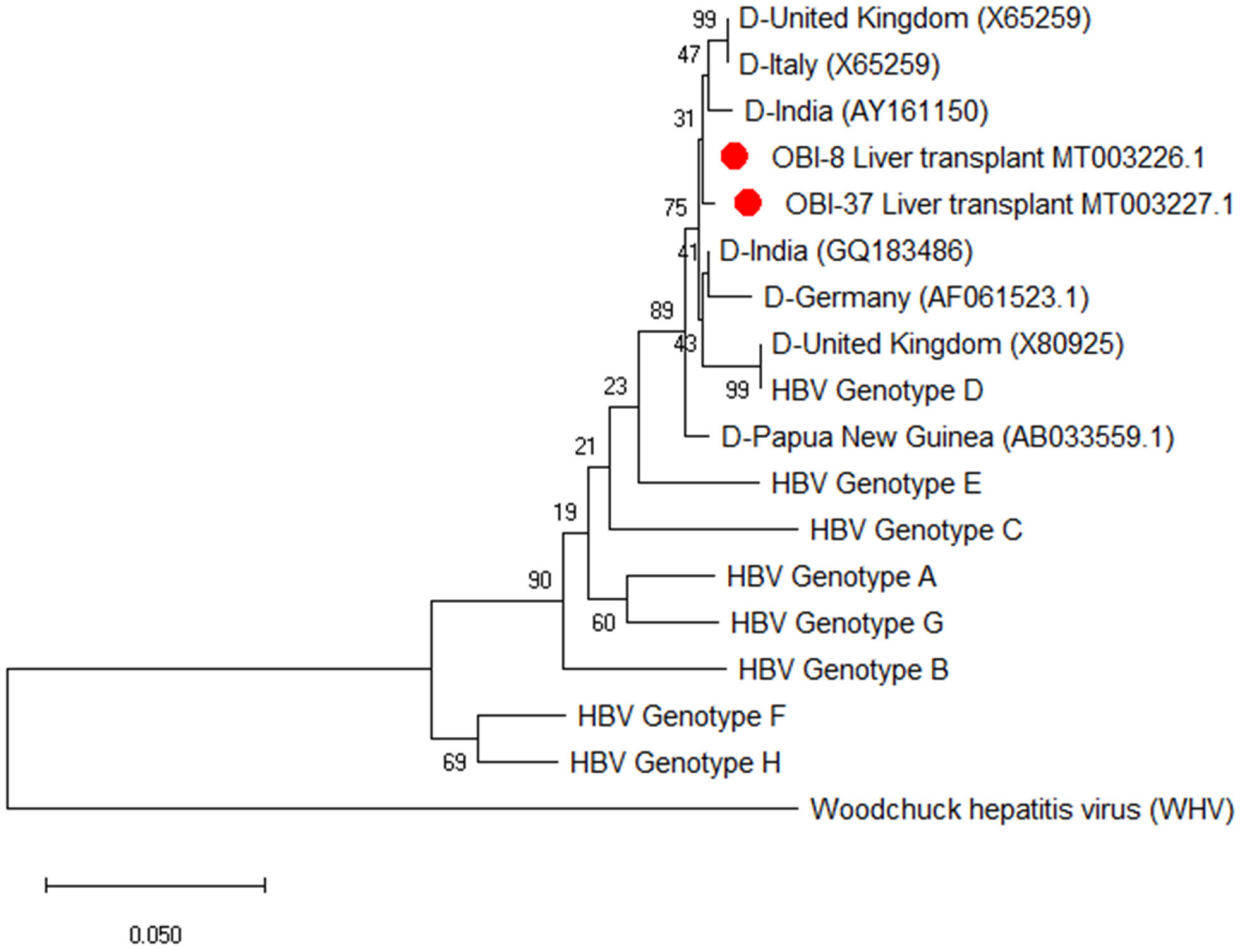
Maximum likelihood phylogenetic tree construction based on the Clustal W alignment of two HBsAg sequences (681‐bp) obtained from Iranian liver transplant patients

**FIGURE 2:**
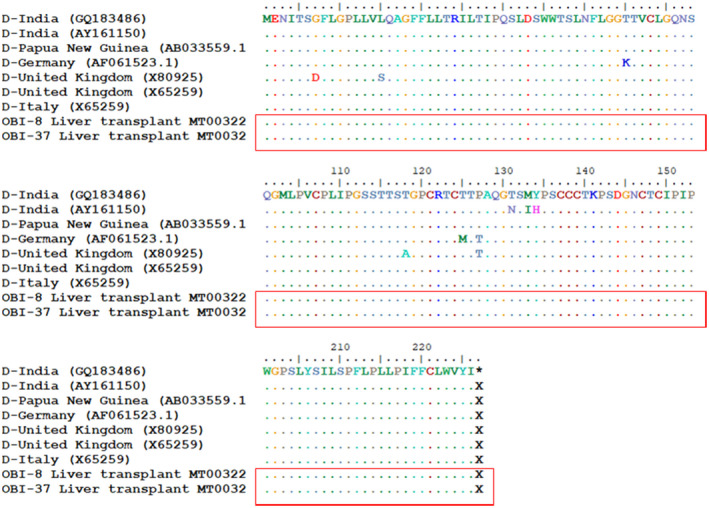
Details of two HBsAg sequences from OBI‐positive cases. Seven HBV genotype D‐matched sequences (namely CQ183486, AY161150, AB0335591, AF061523.1, X80925, X65259, and X65259) were obtained from the NCBI site) to evaluate mutations in the HBsAg region. Amino acid substitutions are shown in different colors by the BioEdit program. Seven top sequences belonged to the HBsAg sequences from a genotype D‐matched isolate.

Of the 97 patients, 57 (58%) and 40 (42%) individuals were male and female, respectively. The participants' age ranged from 15 to 65 years old, with the mean age of 47.66 ± 11.71 for males and 38.72 ± 13.07 for females (*P* = .002). At the time of presentation, no case was positive for HBsAg; however, 24 (25%), 12 (12.3%), and 5 (5.1%) cases were positive for anti‐HBc, anti‐HCV, and anti‐HDV, respectively (Table 1). Moreover, 21 (27%) and 3 (7.5%) males and females were positive for anti‐HBc, respectively. Of the anti‐HCV‐positive cases, 9 (15.7%) and 3 (7.5%) individuals were male and female, respectively. Finally, 4 (7%) and 1 (2.5%) cases of the HDV‐positive patients were male and female, respectively. Two 44 and 45 year‐old males were positive for OBI (2.1%), both of whom were diagnosed by the real‐time and standard PCR methods. Both OBI‐positive samples contained HBV DNA levels <500 copies/mL. The OBI diagnosis was reached 2 years after transplantation. Both cases were positive for anti‐HBc and negative for anti‐HBs, anti‐HCV, and anti‐HDV. Furthermore, HBV‐related cirrhosis was the underlying reason for their liver transplantation. The phylogenetic tree results revealed that both isolates were HBV genotype D and subtype awy2 (Figure 1). In comparison to seven HBV genotype D sequences, the HBsAg gene and also “a” determinant region (amino acid positions 124‐147) revealed no amino acid substitution (Figure 2). Both sequences have been submitted to the GenBank under accession numbers: MT003226‐MT003227. During the follow‐up period (started in 2005), none of the patients, including the OBI‐positive cases, developed the evidence of HBV reactivation.

**TABLE 1:** General characteristics of patients with history of liver disease

Patient Code	Age	Date Link	Date Link	Real time	HBs Ag	HBs Ab	HBc Ab	HBe Ag and HBe Ab	HCV Ab	HDV Ab	Etiology
1	55	1394	2015	‐	‐	3.99	Positive	‐	Positive	‐	HCV cirrhosis
2	60	1393	2014	‐	‐	82	‐	‐	‐	‐	Cryptogenic cirrhosis
3	27	1394	2015	‐	‐	251	‐	‐	‐	‐	Cryptogenic cirrhosis
4	29	1394	2015	‐	‐	0	‐	‐	‐	‐	Autoimmune Hepatitis‐ cirrhosis
5	15	1394	2015	‐	‐	263	‐	‐	‐	‐	Autoimmune Hepatitis‐ cirrhosis
6	33	1394	2015	‐	‐	0	‐	‐	‐	‐	Autoimmune Hepatitis‐ cirrhosis
7	56	1394	2015	‐	‐	0.99	‐	‐	‐	‐	Autoimmune Hepatitis‐ cirrhosis
8	45	1392	2013	Positive	‐	0	Positive	‐	‐	‐	HBV cirrhosis
9	52	1392	2013	‐	‐	1.2	Positive	‐	‐	‐	Hepatitis C ‐ Hepatitis B
10	52	1394	2015	‐	‐	252	Positive	‐	Positive	‐	HCV cirrhosis
11	62	1394	2015	‐	‐	0.11	Positive	‐	‐	‐	Cryptogenic cirrhosis
12	54	1393	2014	‐	‐	0	‐	‐	‐	‐	Cryptogenic cirrhosis
13	41	1393	2014	‐	‐	0	‐	‐	‐	‐	Cryptogenic cirrhosis
14	24	1394	2015	‐	‐	274	‐	‐	‐	‐	Cryptogenic cirrhosis
15	59	1392	2013	‐	‐	68	‐	‐	‐	‐	Cryptogenic cirrhosis
16	37	1384	2005	‐	‐	0	‐	‐	‐	‐	Autoimmune Hepatitis‐ cirrhosis
17	25	1389	2010	‐	‐	301	‐	‐	‐	‐	Autoimmune Hepatitis‐ cirrhosis
18	23	1389	2010	‐	‐	10	‐	‐	‐	‐	Cryptogenic cirrhosis
19	47	1389	2010	‐	‐	0	‐	‐	‐	‐	Cryptogenic cirrhosis
20	46	1393	2014	‐	‐	283	‐	‐	‐	‐	Autoimmune Hepatitis‐ cirrhosis
21	40	1394	2015	‐	‐	0	‐	‐	‐	‐	Autoimmune Hepatitis‐ cirrhosis
22	35	1391	2012	‐	‐	67	‐	‐	‐	‐	Cryptogenic cirrhosis
23	32	1392	2013	‐	‐	23	‐	‐	‐	‐	Budd‐Chiari cirrhosis
24	62	1394	2015	‐	‐	305	Positive	‐	‐	‐	Cryptogenic cirrhosis
25	37	1393	2014	‐	‐	0	‐	‐	‐	‐	Primary sclerosing cholangitis, cirrhosis
26	50	1394	2015	‐	‐	0	‐	‐	Positive	‐	HCV cirrhosis
27	57	1394	2015	‐	‐	48	‐	‐	‐	‐	Cryptogenic cirrhosis
28	65	1394	2015	‐	‐	280	Positive	‐	‐	Positive	HBV cirrhosis
29	50	1391	2012	‐	‐	0	Positive	‐	‐	‐	HBV cirrhosis
	38	1393	2014	‐	‐	0	Positive	‐	‐	Positive	HBV cirrhosis
31	46	1391	2012	‐	‐	20	‐	‐	‐	‐	Cryptogenic cirrhosis
32	63	1393	2014	‐	‐	19	Positive	‐	‐	‐	HBV cirrhosis
33	30	1391	2012	‐	‐	17	‐	‐	‐	‐	Autoimmune Hepatitis‐ cirrhosis
34	21	1393	2014	‐	‐	17	‐	‐	‐	‐	Cryptogenic cirrhosis
35	35	1386	2007	‐	‐	0	‐	‐	‐	‐	Cryptogenic cirrhosis
36	48	1391	2012	‐	‐	0	‐	‐	‐	‐	Cryptogenic cirrhosis
37	44	1392	2013	Positive	‐	0	Positive	‐	‐	‐	HBV cirrhosis
38	21	1392	2013	‐	‐	1.2	‐	‐	‐	‐	Autoimmune Hepatitis‐ cirrhosis
39		1391	2012	‐	‐	1.6	‐	‐	‐	‐	Autoimmune Hepatitis‐ cirrhosis
40	50	1394	2015	‐	‐	104	Positive	‐	‐	‐	Cryptogenic cirrhosis
41	53	1394	2015	‐	‐	53	‐	‐	Positive	‐	HCV cirrhosis
42		1394	2015	‐	‐	79	‐	‐	‐	‐	Cryptogenic cirrhosis
43	52	1392	2013	‐	‐	0.17	Positive	‐	‐	Positive	Cryptogenic cirrhosis
44	60	1389	2010	‐	‐	0	‐	‐	‐	‐	Cryptogenic cirrhosis
45	63	1391	2012	‐	‐	3.8	Positive	‐	‐	Positive	HBV cirrhosis
46	52	1395	2016	‐	‐	32	‐	‐	‐	‐	Cryptogenic cirrhosis
47	50	1394	2015	‐	‐	25	‐	‐	‐	‐	Cryptogenic cirrhosis
48	46	1394	2015	‐	‐	12	‐	‐	Positive	‐	HCV cirrhosis
49	43	1394	2015	‐	‐	0.84	‐	‐	‐	‐	Cryptogenic cirrhosis
50	26	1394	2015	‐	‐	73	‐	‐	‐	‐	Cryptogenic cirrhosis
51	42	1393	2014	‐	‐	1.3	‐	‐	‐	‐	Cryptogenic cirrhosis
52	63	1395	2016	‐	‐	3.7	‐	‐	‐	‐	Cryptogenic cirrhosis
53	27	1394	2015	‐	‐	126	‐	‐	‐	‐	Primary sclerosing cholangitis, cirrhosis
54	24	1385	2006	‐	‐	254	‐	‐	‐	‐	Cryptogenic cirrhosis
55	55	1389	2010	‐	‐	0.5	‐	‐	‐	‐	Cryptogenic cirrhosis
56	63	1392	2013	‐	‐	0.2	‐	‐	‐	‐	Cryptogenic cirrhosis
57	25	1391	2012	‐	‐	6	‐	‐	‐	‐	Cryptogenic cirrhosis
58	60	1394	2015	‐	‐	11	Positive	‐	‐	‐	Cryptogenic cirrhosis
59	46	1392	2013	‐	‐	4/0	‐	‐	‐	‐	Cryptogenic cirrhosis
60	38	1391	2012	‐	‐	17/3	‐	‐	‐	‐	Cryptogenic cirrhosis
61	31	1390	2011	‐	‐	19	‐	‐	‐	‐	Autoimmune Hepatitis‐ cirrhosis
62	55	1393	2014	‐	‐	9/2	‐	‐	‐	‐	Cryptogenic cirrhosis
63		1394	2015	‐	‐	135	‐	‐	‐	‐	Cryptogenic cirrhosis
64	53	1395	2016	‐	‐	258	Positive	‐	‐	‐	HBV cirrhosis
65		1387	2008	‐	‐	4	‐	‐	‐	‐	Cryptogenic cirrhosis
66		1394	2015	‐	‐	4	‐	‐	‐	‐	Cryptogenic cirrhosis
67	40	1394	2015	‐	‐	13	‐	‐	‐	‐	Autoimmune Hepatitis‐ cirrhosis
68	42	1391	2012	‐	‐	5/3	Positive	‐	‐	‐	HBV cirrhosis
69		1388	2009	‐	‐	4/2	‐	‐	Positive	‐	HCV cirrhosis
70		1392	2013	‐	‐	34	‐	‐	‐	‐	Cryptogenic cirrhosis
71		1389	2010	‐	‐	7/0	Positive	‐	‐	‐	HBV cirrhosis
72	24	1391	2012	‐	‐	211	‐	‐	‐	‐	Cryptogenic cirrhosis
73		1395	2016	‐	‐	1	‐	‐	‐	‐	Cryptogenic cirrhosis
74		1394	2015	‐	‐	9/0	‐	‐	Positive	‐	HCV cirrhosis
75	60	1393	2014	‐	‐	273	Positive	‐	‐	‐	Cryptogenic cirrhosis
76		‐	‐	‐	‐	13	Positive	‐	‐	‐	Cryptogenic cirrhosis
77	55	1393	2014	‐	‐	7/6	‐	‐	‐	‐	Autoimmune Hepatitis‐ cirrhosis
78	57	1393	2014	‐	‐	17	Positive	‐	‐	‐	Cryptogenic cirrhosis
79	45	1395	2016	‐	‐	2/1	‐	‐	‐	‐	Fulminant Hepatitis
80	37	1394	2015	‐	‐	40	Positive	‐	‐	‐	HBV cirrhosis
81	55	1388	2009	‐	‐	9/1	‐	‐	Positive	‐	HCV cirrhosis
82	44	1394	2015	‐	‐	2/4	Positive	‐	‐	‐	Cryptogenic cirrhosis
83	40	1389	2010	‐	‐	11	‐	‐	‐	Positive	HBV cirrhosis
84	37	1392	2013	‐	‐	48	‐	‐	‐	‐	Cryptogenic cirrhosis
85	55	1390	2011	‐	‐	1/8	Positive	‐	Positive	‐	HCV cirrhosis
86	57	1393	2014	‐	‐	8/0	‐	‐	Positive	‐	HCV cirrhosis
87	44	1393	2014	‐	‐	7/0	‐	‐	Positive	‐	HCV cirrhosis
88	54	1395	2016	‐	‐	4/0	‐	‐	Positive	‐	HCV cirrhosis
89	40	1395	2016	‐	‐	1/1	‐	‐	‐	‐	Autoimmune Hepatitis‐ cirrhosis
90	40	1388	2009	‐	‐	3/0	‐	‐	‐	‐	Autoimmune Hepatitis‐ cirrhosis
91	32	‐	‐	‐	‐	5/4	‐	‐	‐	‐	Primary sclerosing cholangitis, cirrhosis
92		‐	‐	‐	‐	171	‐	‐	‐	‐	HBV cirrhosis
93	30	1393	2014	‐	‐	6/8	‐	‐	‐	‐	Cryptogenic cirrhosis
94		1395	2016	‐	‐	22	‐	‐	‐	‐	Cryptogenic cirrhosis
95	56	1393	2014	‐	‐	8/4	‐	‐	‐	‐	Cryptogenic cirrhosis
96	30	1391	2012	‐	‐	4	‐	‐	‐	‐	Autoimmune Hepatitis‐ cirrhosis
97	51	1394	2015	‐	‐	5/7	‐	‐	‐	‐	Cryptogenic cirrhosis

Etiology of liver transplantation in patients studied case	
The cause of liver Transplantation	Number (percent)
Cryptogenic cirrhosis	49 (50.5)
HBV‐ cirrhosis	14 (14.4)
HCV‐ cirrhosis	13 (13.4)
Autoimmune hepatitis cirrhosis	17 (17.5)
Fulminant hepatitis cirrhosis	1 (1)
Budd‐Chiari cirrhosis	1 (1)
Primary sclerosing cholangitis (PSC) cirrhosis	3 (3)

## 4 DISCUSSION

One of the main clinical implications of OBI is usually observed in the setting of liver transplantation. In particular, livers from donors with OBI carry a risk of HBV transmission, with infection arousing in 25%‐95% of the liver grafts donated from patients being HBsAg negative and anti‐HBc positive.^2^ The present study documented the relatively low prevalence of OBI among the Iranian liver transplant recipients (2.1%). OBI is one of the causes of cryptogenic cirrhosis, and there is 38% OBI among Iranian patients diagnosed with this type of clinical setting.^15^ Another Iranian study reported 14% OBI in this clinical setting.^16^ However, using two different sensitive molecular approaches, we found no symptom of HBV DNA in 49 patients diagnosed with cryptogenic cirrhosis before LT. It is suggested that no cccDNA exists after LT and the resection of the liver with the lack of the primary source for HBV replication. However, cccDNA could have existed in other extrahepatic sources such as PBMC.^17^

Two OBI patients in the present study were positive for anti‐HBc and negative for anti‐HBs. The hypothesis behind the low prevalence of OBI among Iranian liver transplant recipients remains elusive. However, several factors have been attributed to the evolution of HBV around the globe, of which the HBV genetic variations with the highest frequency have been the most underlying factor. Several HBV mutations within the “a” determinant of HBsAg have been reported in association with OBI.^18^ However, in the present study, no mutation was found in this regard in any patient. According to the findings released by the Iran Hepatitis Network, all Iranian HBV‐positive carriers contained genotype D, and 61.9% of the Iranian chronic HBV carriers had mutations in their HBsAg genes.^19^ However, of 542 amino acid variations, 404 (74.5%) cases were naturally occurring mutations. This unique pattern has not been reported for other HBV‐infected populations yet.

On the other hand, the HBV genotypes B and C are prevalent among Southeast Asian chronic HBV populations.^20^ Those genotypes contained the highest heterogeneity, including sub‐genotypes (inter‐host variations) and mutational drifts (intra‐host variations).^21^ Nevertheless, the HLA‐related genetic background of these populations contributed to a high HBV chronicity rate and the relevant consequences of the HBV infection, including cirrhosis and HCC.^22^, ^23^

## 5 CONCLUSIONS

The prevalence of OBI among Iranian patients referred to one of the two Iranian referral centers for liver transplantation was relatively low. Future longitudinal studies are required to employ a larger sample size to explore the significance of this clinical finding, including the reactivation of cryptic HBV DNA, in liver transplant subjects.

## AUTHOR CONTRIBUTIONS

Study concept, design, and drafting of the manuscript: Seyed Mohammad Jazayeri and Mohsen Nasiri toosi; Acquisition of data and experimental analysis: Azam Khamseh, Vahdat Poortahmasebi, Azam Ghaziasadi, Saber Soltani, and Saeid Ghorbani; Interpretation of data: Ali Jafarian, Mehdi Norouzi and Narges Eslami.

## CONFLICT OF INTEREST

None of the authors declared any conflict of interest.

## Supporting information


Appendix S1

## Data Availability

All data related to this work are available from the corresponding author upon reasonable request.
