# Supplementary material for: Characterization of occult hepatitis B infection among Iranian liver transplant recipients
Source: J Clin Lab Anal. 2022 Sep 9;36(10):e24614. doi: 10.1002/jcla.24614 (PMC9551123; doi:10.1002/jcla.24614)
Supplement: Supplementary file 1 — Appendix S1 [file JCLA-36-e24614-s001.docx]

**Table of general characteristics of patients with history of liver disease**

| **Etiology** | **HDV Ab** | **HCV Ab** | **HBe Ag& HBe Ab** | **HBc Ab** | **HBs Ab** | **HBs Ag** | **Real time** | **Date Link** | **Date Link** | **Age** | **Patient Code** |
| --- | --- | --- | --- | --- | --- | --- | --- | --- | --- | --- | --- |
| **HCV cirrhosis** | **-** | **Positive** | **-** | **Positive** | **3.99** | **-** | **-** | **2015** | **1394** | **55** | **1** |
| **Cryptogenic cirrhosis** | **-** | **-** | **-** | **-** | **82** | **-** | **-** | **2014** | **1393** | **60** | **2** |
| **Cryptogenic cirrhosis** | **-** | **-** | **-** | **-** | **251** | **-** | **-** | **2015** | **1394** | **27** | **3** |
| **Autoimmune Hepatitis- cirrhosis** | **-** | **-** | **-** | **-** | **0** | **-** | **-** | **2015** | **1394** | **29** | **4** |
| **Autoimmune Hepatitis- cirrhosis** | **-** | **-** | **-** | **-** | **263** | **-** | **-** | **2015** | **1394** | **15** | **5** |
| **Autoimmune Hepatitis- cirrhosis** | **-** | **-** | **-** | **-** | **0** | **-** | **-** | **2015** | **1394** | **33** | **6** |
| **Autoimmune Hepatitis- cirrhosis** | **-** | **-** | **-** | **-** | **0.99** | **-** | **-** | **2015** | **1394** | **56** | **7** |
| **HBV cirrhosis** | **-** | **-** | **-** | **Positive** | **0** | **-** | **Positive** | **2013** | **1392** | **45** | **8** |
| **Hepatitis C - Hepatitis B** | **-** | **-** | **-** | **Positive** | **1.2** | **-** | **-** | **2013** | **1392** | **52** | **9** |
| **HCV cirrhosis** | **-** | **Positive** | **-** | **Positive** | **252** | **-** | **-** | **2015** | **1394** | **52** | **10** |
| **Cryptogenic cirrhosis** | **-** | **-** | **-** | **Positive** | **0.11** | **-** | **-** | **2015** | **1394** | **62** | **11** |
| **Cryptogenic cirrhosis** | **-** | **-** | **-** | **-** | **0** | **-** | **-** | **2014** | **1393** | **54** | **12** |
| **Cryptogenic cirrhosis** | **-** | **-** | **-** | **-** | **0** | **-** | **-** | **2014** | **1393** | **41** | **13** |
| **Cryptogenic cirrhosis** | **-** | **-** | **-** | **-** | **274** | **-** | **-** | **2015** | **1394** | **24** | **14** |
| **Cryptogenic cirrhosis** | **-** | **-** | **-** | **-** | **68** | **-** | **-** | **2013** | **1392** | **59** | **15** |
| **Autoimmune Hepatitis- cirrhosis** | **-** | **-** | **-** | **-** | **0** | **-** | **-** | **2005** | **1384** | **37** | **16** |
| **Autoimmune Hepatitis- cirrhosis** | **-** | **-** | **-** | **-** | **301** | **-** | **-** | **2010** | **1389** | **25** | **17** |
| **Cryptogenic cirrhosis** | **-** | **-** | **-** | **-** | **10** | **-** | **-** | **2010** | **1389** | **23** | **18** |
| **Cryptogenic cirrhosis** | **-** | **-** | **-** | **-** | **0** | **-** | **-** | **2010** | **1389** | **47** | **19** |
| **Autoimmune Hepatitis- cirrhosis** | **-** | **-** | **-** | **-** | **283** | **-** | **-** | **2014** | **1393** | **46** | **20** |
| **Autoimmune Hepatitis- cirrhosis** | **-** | **-** | **-** | **-** | **0** | **-** | **-** | **2015** | **1394** | **40** | **21** |
| **Cryptogenic cirrhosis** | **-** | **-** | **-** | **-** | **67** | **-** | **-** | **2012** | **1391** | **35** | **22** |
| **Budd-Chiari cirrhosis** | **-** | **-** | **-** | **-** | **23** | **-** | **-** | **2013** | **1392** | **32** | **23** |
| **Cryptogenic cirrhosis** | **-** | **-** | **-** | **Positive** | **305** | **-** | **-** | **2015** | **1394** | **62** | **24** |
| **Primary sclerosing cholangitis, cirrhosis** | **-** | **-** | **-** | **-** | **0** | **-** | **-** | **2014** | **1393** | **37** | **25** |
| **HCV cirrhosis** | **-** | **Positive** | **-** | **-** | **0** | **-** | **-** | **2015** | **1394** | **50** | **26** |
| **Cryptogenic cirrhosis** | **-** | **-** | **-** | **-** | **48** | **-** | **-** | **2015** | **1394** | **57** | **27** |
| **HBV cirrhosis** | **Positive** | **-** | **-** | **Positive** | **280** | **-** | **-** | **2015** | **1394** | **65** | **28** |
| **HBV cirrhosis** | **-** | **-** | **-** | **Positive** | **0** | **-** | **-** | **2012** | **1391** | **50** | **29** |
| **HBV cirrhosis** | **Positive** | **-** | **-** | **Positive** | **0** | **-** | **-** | **2014** | **1393** | **38** |  |
| **Cryptogenic cirrhosis** | **-** | **-** | **-** | **-** | **20** | **-** | **-** | **2012** | **1391** | **46** | **31** |
| **HBV cirrhosis** | **-** | **-** | **-** | **Positive** | **19** | **-** | **-** | **2014** | **1393** | **63** | **32** |
| **Autoimmune Hepatitis- cirrhosis** | **-** | **-** | **-** | **-** | **17** | **-** | **-** | **2012** | **1391** | **30** | **33** |
| **Cryptogenic cirrhosis** | **-** | **-** | **-** | **-** | **17** | **-** | **-** | **2014** | **1393** | **21** | **34** |
| **Cryptogenic cirrhosis** | **-** | **-** | **-** | **-** | **0** | **-** | **-** | **2007** | **1386** | **35** | **35** |
| **Cryptogenic cirrhosis** | **-** | **-** | **-** | **-** | **0** | **-** | **-** | **2012** | **1391** | **48** | **36** |
| **HBV cirrhosis** | **-** | **-** | **-** | **Positive** | **0** | **-** | **Positive** | **2013** | **1392** | **44** | **37** |
| **Autoimmune Hepatitis- cirrhosis** | **-** | **-** | **-** | **-** | **1.2** | **-** | **-** | **2013** | **1392** | **21** | **38** |
| **Autoimmune Hepatitis- cirrhosis** | **-** | **-** | **-** | **-** | **1.6** | **-** | **-** | **2012** | **1391** |  | **39** |
| **Cryptogenic cirrhosis** | **-** | **-** | **-** | **Positive** | **104** | **-** | **-** | **2015** | **1394** | **50** | **40** |
| **HCV cirrhosis** | **-** | **Positive** | **-** | **-** | **53** | **-** | **-** | **2015** | **1394** | **53** | **41** |
| **Cryptogenic cirrhosis** | **-** | **-** | **-** | **-** | **79** | **-** | **-** | **2015** | **1394** |  | **42** |
| **Cryptogenic cirrhosis** | **Positive** | **-** | **-** | **Positive** | **0.17** | **-** | **-** | **2013** | **1392** | **52** | **43** |
| **Cryptogenic cirrhosis** | **-** | **-** | **-** | **-** | **0** | **-** | **-** | **2010** | **1389** | **60** | **44** |
| **HBV cirrhosis** | **Positive** | **-** | **-** | **Positive** | **3.8** | **-** | **-** | **2012** | **1391** | **63** | **45** |
| **Cryptogenic cirrhosis** | **-** | **-** | **-** | **-** | **32** | **-** | **-** | **2016** | **1395** | **52** | **46** |
| **Cryptogenic cirrhosis** | **-** | **-** | **-** | **-** | **25** | **-** | **-** | **2015** | **1394** | **50** | **47** |
| **HCV cirrhosis** | **-** | **Positive** | **-** | **-** | **12** | **-** | **-** | **2015** | **1394** | **46** | **48** |
| **Cryptogenic cirrhosis** | **-** | **-** | **-** | **-** | **0.84** | **-** | **-** | **2015** | **1394** | **43** | **49** |
| **Cryptogenic cirrhosis** | **-** | **-** | **-** | **-** | **73** | **-** | **-** | **2015** | **1394** | **26** | **50** |
| **Cryptogenic cirrhosis** | **-** | **-** | **-** | **-** | **1.3** | **-** | **-** | **2014** | **1393** | **42** | **51** |
| **Cryptogenic cirrhosis** | **-** | **-** | **-** | **-** | **3.7** | **-** | **-** | **2016** | **1395** | **63** | **52** |
| **Primary sclerosing cholangitis, cirrhosis** | **-** | **-** | **-** | **-** | **126** | **-** | **-** | **2015** | **1394** | **27** | **53** |
| **Cryptogenic cirrhosis** | **-** | **-** | **-** | **-** | **254** | **-** | **-** | **2006** | **1385** | **24** | **54** |
| **Cryptogenic cirrhosis** | **-** | **-** | **-** | **-** | **0.5** | **-** | **-** | **2010** | **1389** | **55** | **55** |
| **Cryptogenic cirrhosis** | **-** | **-** | **-** | **-** | **0.2** | **-** | **-** | **2013** | **1392** | **63** | **56** |
| **Cryptogenic cirrhosis** | **-** | **-** | **-** | **-** | **6** | **-** | **-** | **2012** | **1391** | **25** | **57** |
| **Cryptogenic cirrhosis** | **-** | **-** | **-** | **Positive** | **11** | **-** | **-** | **2015** | **1394** | **60** | **58** |
| **Cryptogenic cirrhosis** | **-** | **-** | **-** | **-** | **4/0** | **-** | **-** | **2013** | **1392** | **46** | **59** |
| **Cryptogenic cirrhosis** | **-** | **-** | **-** | **-** | **17/3** | **-** | **-** | **2012** | **1391** | **38** | **60** |
| **Autoimmune Hepatitis- cirrhosis** | **-** | **-** | **-** | **-** | **19** | **-** | **-** | **2011** | **1390** | **31** | **61** |
| **Cryptogenic cirrhosis** | **-** | **-** | **-** | **-** | **9/2** | **-** | **-** | **2014** | **1393** | **55** | **62** |
| **Cryptogenic cirrhosis** | **-** | **-** | **-** | **-** | **135** | **-** | **-** | **2015** | **1394** |  | **63** |
| **HBV cirrhosis** | **-** | **-** | **-** | **Positive** | **258** | **-** | **-** | **2016** | **1395** | **53** | **64** |
| **Cryptogenic cirrhosis** | **-** | **-** | **-** | **-** | **4** | **-** | **-** | **2008** | **1387** |  | **65** |
| **Cryptogenic cirrhosis** | **-** | **-** | **-** | **-** | **4** | **-** | **-** | **2015** | **1394** |  | **66** |
| **Autoimmune Hepatitis- cirrhosis** | **-** | **-** | **-** | **-** | **13** | **-** | **-** | **2015** | **1394** | **40** | **67** |
| **HBV cirrhosis** | **-** | **-** | **-** | **Positive** | **5/3** | **-** | **-** | **2012** | **1391** | **42** | **68** |
| **HCV cirrhosis** | **-** | **Positive** | **-** | **-** | **4/2** | **-** | **-** | **2009** | **1388** |  | **69** |
| **Cryptogenic cirrhosis** | **-** | **-** | **-** | **-** | **34** | **-** | **-** | **2013** | **1392** |  | **70** |
| **HBV cirrhosis** | **-** | **-** | **-** | **Positive** | **7/0** | **-** | **-** | **2010** | **1389** |  | **71** |
| **Cryptogenic cirrhosis** | **-** | **-** | **-** | **-** | **211** | **-** | **-** | **2012** | **1391** | **24** | **72** |
| **Cryptogenic cirrhosis** | **-** | **-** | **-** | **-** | **1** | **-** | **-** | **2016** | **1395** |  | **73** |
| **HCV cirrhosis** | **-** | **Positive** | **-** | **-** | **9/0** | **-** | **-** | **2015** | **1394** |  | **74** |
| **Cryptogenic cirrhosis** | **-** | **-** | **-** | **Positive** | **273** | **-** | **-** | **2014** | **1393** | **60** | **75** |
| **Cryptogenic cirrhosis** | **-** | **-** | **-** | **Positive** | **13** | **-** | **-** | **-** | **-** |  | **76** |
| **Autoimmune Hepatitis- cirrhosis** | **-** | **-** | **-** | **-** | **7/6** | **-** | **-** | **2014** | **1393** | **55** | **77** |
| **Cryptogenic cirrhosis** | **-** | **-** | **-** | **Positive** | **17** | **-** | **-** | **2014** | **1393** | **57** | **78** |
| **Fulminant Hepatitis** | **-** | **-** | **-** | **-** | **2/1** | **-** | **-** | **2016** | **1395** | **45** | **79** |
| **HBV cirrhosis** | **-** | **-** | **-** | **Positive** | **40** | **-** | **-** | **2015** | **1394** | **37** | **80** |
| **HCV cirrhosis** | **-** | **Positive** | **-** | **-** | **9/1** | **-** | **-** | **2009** | **1388** | **55** | **81** |
| **Cryptogenic cirrhosis** | **-** | **-** | **-** | **Positive** | **2/4** | **-** | **-** | **2015** | **1394** | **44** | **82** |
| **HBV cirrhosis** | **Positive** | **-** | **-** | **-** | **11** | **-** | **-** | **2010** | **1389** | **40** | **83** |
| **Cryptogenic cirrhosis** | **-** | **-** | **-** | **-** | **48** | **-** | **-** | **2013** | **1392** | **37** | **84** |
| **HCV cirrhosis** | **-** | **Positive** | **-** | **Positive** | **1/8** | **-** | **-** | **2011** | **1390** | **55** | **85** |
| **HCV cirrhosis** | **-** | **Positive** | **-** | **-** | **8/0** | **-** | **-** | **2014** | **1393** | **57** | **86** |
| **HCV cirrhosis** | **-** | **Positive** | **-** | **-** | **7/0** | **-** | **-** | **2014** | **1393** | **44** | **87** |
| **HCV cirrhosis** | **-** | **Positive** | **-** | **-** | **4/0** | **-** | **-** | **2016** | **1395** | **54** | **88** |
| **Autoimmune Hepatitis- cirrhosis** | **-** | **-** | **-** | **-** | **1/1** | **-** | **-** | **2016** | **1395** | **40** | **89** |
| **Autoimmune Hepatitis- cirrhosis** | **-** | **-** | **-** | **-** | **3/0** | **-** | **-** | **2009** | **1388** | **40** | **90** |
| **Primary sclerosing cholangitis, cirrhosis** | **-** | **-** | **-** | **-** | **5/4** | **-** | **-** | **-** | **-** | **32** | **91** |
| **HBV cirrhosis** | **-** | **-** | **-** | **-** | **171** | **-** | **-** | **-** | **-** |  | **92** |
| **Cryptogenic cirrhosis** | **-** | **-** | **-** | **-** | **6/8** | **-** | **-** | **2014** | **1393** | **30** | **93** |
| **Cryptogenic cirrhosis** | **-** | **-** | **-** | **-** | **22** | **-** | **-** | **2016** | **1395** |  | **94** |
| **Cryptogenic cirrhosis** | **-** | **-** | **-** | **-** | **8/4** | **-** | **-** | **2014** | **1393** | **56** | **95** |
| **Autoimmune Hepatitis- cirrhosis** | **-** | **-** | **-** | **-** | **4** | **-** | **-** | **2012** | **1391** | **30** | **96** |
| **Cryptogenic cirrhosis** | **-** | **-** | **-** | **-** | **5/7** | **-** | **-** | **2015** | **1394** | **51** | **97** |

**Etiology of liver transplantation in patients Studied case**

| **Number (percent)** | **The cause of liver Transplantation** |
| --- | --- |
| **49 (50.5)** | **Cryptogenic cirrhosis** |
| **14 (14.4)** | **HBV- cirrhosis** |
| **13 (13.4)** | **HCV- cirrhosis** |
| **17 (17.5)** | **Autoimmune hepatitis cirrhosis** |
| **1 (1)** | **Fulminant hepatitis cirrhosis** |
| **1 (1)** | **Budd-Chiari cirrhosis** |
| **3 (3)** | **Primary sclerosing cholangitis (PSC) cirrhosis** |
